# Mechanisms Behind the Indirect Impact of Metabolic Regulators on Virulence Factor Production in Staphylococcus aureus

**DOI:** 10.1128/spectrum.02063-22

**Published:** 2022-07-05

**Authors:** Amelia C. Stephens, Lance R. Thurlow, Anthony R. Richardson

**Affiliations:** a Department of Microbiology and Molecular Genetics, University of Pittsburgh, Pittsburgh, Pennsylvania, USA; University of Florida College of Dentistry

**Keywords:** *Staphylococcus aureus*, carbon metabolism, metabolic regulation, metabolism, quorum sensing, virulence factors, virulence regulation

## Abstract

Staphylococcus aureus is a human skin pathogen capable of causing invasive infections in many tissues in the human body. The host of virulence factors, such as toxins and proteases, available to S. aureus contribute to its diverse disease presentations. The majority of these virulence factors are under the control of the Agr quorum sensing system. The interaction between the Agr system and some well-established metabolic regulators has long been noted, but no mechanism has been provided as to these indirect interactions. In this study, we examine the connection between Agr and CcpA, a regulator of central carbon metabolism with a known positive impact on Agr function. We further investigated the interaction of Agr and CodY, a regulator of amino acid metabolism and a member of the stringent response with a known negative impact on Agr function. We show that though there are alterations in intracellular amino acid levels in each of these mutants that are consistent with their effect on Agr, there does not seem to be a direct impact on the translation of the Agr system itself that contributes to the altered expression observed in these mutants. Given the changes in cellular metabolism in a *ΔccpA* mutant, we find reduced levels of intracellular ATP even in the presence of glucose. This reduction in ATP, combined with the reduced affinity of the AgrC sensor kinase for ATP, explains the reduction in Agr activity long observed in *ΔccpA* strains.

**IMPORTANCE** The human pathogen Staphylococcus aureus produces a great number of virulence factors that contribute to the pathogen’s ability to cause dangerous, invasive infections. Understanding the full scope of the regulation of these virulence factors can provide us with new information about how to target virulence factor production. For years, researchers in the field have observed an impact of metabolic regulators on virulence factor production with no mechanistic explanation. Here, we describe the role of two of these regulators, CcpA and CodY, in virulence factor expression and provide evidence of indirect mechanisms contributing to the control of the Agr system and virulence factor production by these two metabolic regulators. Our study sheds light on the interplay between metabolism and virulence in S. aureus and provides an explanation as to how these concepts are linked.

## INTRODUCTION

Staphylococcus aureus is a Gram-positive bacterial pathogen that typically colonizes the skin and nares of heathy humans (1, 2). In some cases, however, S. aureus can cause manageable disease such as skin and soft tissue infections (SSTIs); it can also progress to more invasive and difficult to manage presentations, such as endocarditis, osteomyelitis, and bacteremia (3–5). Invasive disease often occurs after the skin barrier or mucosal surface has been breached, allowing S. aureus access to a more nutritionally rich environment, especially serum glucose (6). Once in this environment, S. aureus is provided with the benefit of more nutrients, increased growth, and enhanced virulence factor production, allowing S. aureus the chance to establish an active infection.

Quorum sensing is an important aspect of infection for most bacterial species. It involves the ability of the bacteria to sense the cell density of its own species in the immediate area, as well as the presence of other bacterial species. In S. aureus, quorum sensing is mediated by the accessory gene regulatory (Agr) system (7). This system consists of the *AgrBDCA* locus. AgrD, a small peptide, is processed and secreted by AgrB and released into the extracellular environment as auto-inducing peptide, or AIP. AIP signals back to the bacteria in the vicinity by binding to the AgrC sensor kinase, which autophosphorylates and passes that phosphate on to the AgrA response regulator. AgrA then engages transcription at certain promoters, including the Agr locus itself and the neighboring RNAIII promoter. RNAIII is a ~1kb sRNA that encodes the toxin *hld*, but also has pleiotropic effects on the transcript levels of many different virulence factors. These include toxins (alpha-toxin and leukotoxins) and proteases (e.g., aureolysin) capable of causing severe tissue damage, which have long been established as important for S. aureus infection (8–10). The regulation of these virulence factors has been extensively studied, and a general understanding of regulation of the Agr system is widely accepted. However, some aspects of regulation are still unknown, including how metabolic regulators interact with the Agr system.

CcpA (carbon catabolite protein A) is a vital transcriptional regulator for carbon catabolite repression (CCR), the regulation scheme bacteria use to optimize which carbon source they utilize (11). For S. aureus, the carbon source optimized by CCR is glucose. Glucose transporters and glycolysis genes are upregulated by CcpA, and genes relating to the catabolism of other carbon sources (such as amino acids) are repressed. However, in S. aureus, CcpA has been shown to activate genes outside of its canonical regulon. For instance, it has a positive indirect effect on *ldh1*, which is also important for infection (12). This effect only occurs when in the presence of glucose; a *ΔccpA* mutant consuming amino acids does not exhibit this regulation. Additionally, CcpA has a positive impact of Agr function (13–15). Again, this effect is dependent on glucose and is indirect. Despite a virulence defect in *ΔccpA* being reported as early as 2006, no explanation of the mechanism behind decreased Agr activity in *ΔccpA* strains has been provided.

CcpA is not the only metabolic regulator with an effect on Agr function. CodY has been reported to have an indirect negative effect on Agr function, with only speculation as to how this effect is exerted (16–19). CodY responds to levels of branch chain amino acids (BCAAs) and intracellular GTP, becoming activated by low BCAA or GTP levels (20). One way GTP is exhausted is through insufficient amino acid concentrations leading to high levels of uncharged tRNA, activating the stringent response and the concomitant synthesis of ppGpp(p), resulting in lowered GTP levels (21, 22). BCAAs are the most commonly encoded amino acids in most genomes, and their abundance is tightly linked to the overall amino acid content of the cell. Once reduced BCAAs or GTP levels are sensed by CodY, it becomes inactive and derepresses genes related to amino acid synthesis and transport, aiming to increase intracellular amino acid concentrations (21, 22). A Δ*codY* mutant has been shown to have increased RNAIII levels and downstream toxin levels, indicating that the absence of CodY regulation somehow increases Agr signaling and activation (19). While no explanation has been proven, it has been theorized that increased intracellular amino acid pools in a Δ*codY* mutant due to increased synthesis and transport could result in improved translation of common transcripts in the cell, including *agrBDCA*, resulting in increased Agr signaling and activity (18).

With increasing rates of methicillin-resistant S. aureus (MRSA) infections in the United States (23), understanding the different mechanisms S. aureus uses to control Agr activity and the subsequent production of virulence factors is vital. The dominant MRSA clone circulating in the U.S. since the early 2000s is the USA300 clone, which exhibits hyperexpression of virulence factors for unknown reasons (24–28). Understanding any interactions between virulence and the metabolic state of S. aureus could be key to developing new ways to target MRSA infections. One aspect of this interaction is the poor affinity of the response regulator AgrC to ATP, which exhibits a K_M_ ~10× lower than that of a typical sensor kinase (29). This makes the Agr system highly dependent on the energy state of the cell. To this end, the effect of serum glucose on S. aureus disease outcomes has been increasingly appreciated (30). Specifically, increased serum glucose present in individuals with diabetes presents a more abundant energy source to S. aureus infections, allowing the bacteria to increase glycolytic flux and, in turn, cellular ATP levels. This results in increased levels of virulence factor production per bacterium in diabetic mice compared to nondiabetic mice. These diabetic animals experienced overall worsened infections, and these infections were mitigated when key glycolytic genes were knocked out in S. aureus. While that study demonstrated how ATP is linked to Agr and glycolysis, this study aimed to go one step further, and investigate how the genetic regulators of metabolism link to Agr activation as well.

## RESULTS

### A *ΔccpA* strain is attenuated in a diabetes mouse model.

**FIG 1:**
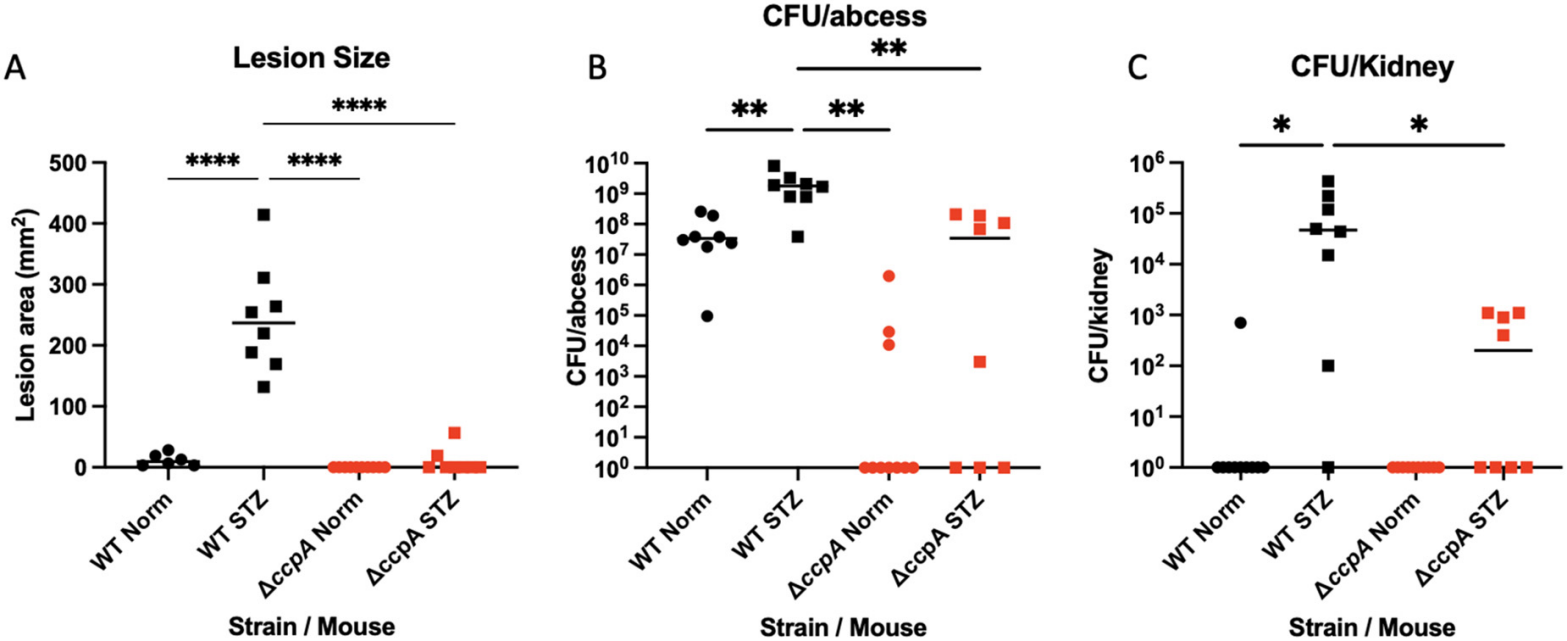
Deletion of *ccpA* results in severely attenuated infections in both normal and diabetic animals. Untreated and STZ treated mice were subcutaneously infected with 1 × 10^7^ CFU WT S. aureus LAC and the isogenic Δ*ccpA* mutant (*n* = 8 for each category). STZ treated mice infected with WT LAC showed larger lesion area (A) and higher bacterial burdens in the abscess and in the kidney (B, C). Mice infected with the Δ*ccpA* mutant exhibited attenuated infection and lowered metrics in all categories (A–C) in both untreated and STZ treated animals. Statistics: unpaired *t* tests between groups *, *P* < 0.05; ****, *P* < .0001.

We infected wild-type (WT) C57J/B6 mice subcutaneously with 10^7^ CFU WT S. aureus LAC and the isogenic *ΔccpA* strain, and mice were monitored daily for weight loss over 7 days. At day 7, mice were sacrificed and analyzed for lesion size and CFU burden at the site of infection and in peripheral organs. Our results show that the *ΔccpA* strain was attenuated in this infection model compared to WT LAC (Fig. 1), displaying decreased lesion area and CFU in both the abscess and disseminated tissues. We also conducted identical infections on mice treated with streptozotocin (STZ) to induce an insulin-dependent diabetic state (via killing of pancreatic β-cells). In this model, which has been characterized as being more susceptible to LAC SSTIs (30), we found significant attenuation of the *ΔccpA* strain in all measures analyzed (Fig. 1). We note that the attenuation seen in the animals infected with the *ΔccpA* strain is more severe than with any other glycolytic or *ΔagrA* mutant tested in our lab (30), with full clearance of the bacteria from ~50% of the infected mice and very low bacterial burden in the others. The abscess also never fully ruptures and becomes necrotic, indicating a much less severe infection than WT infected animals. This indicated to us that CcpA is key for infection, even in conditions where the host is immunocompromised and there is excess glucose available to S. aureus.

### The *ΔccpA* strain has reduced Agr activity.

**FIG 2:**
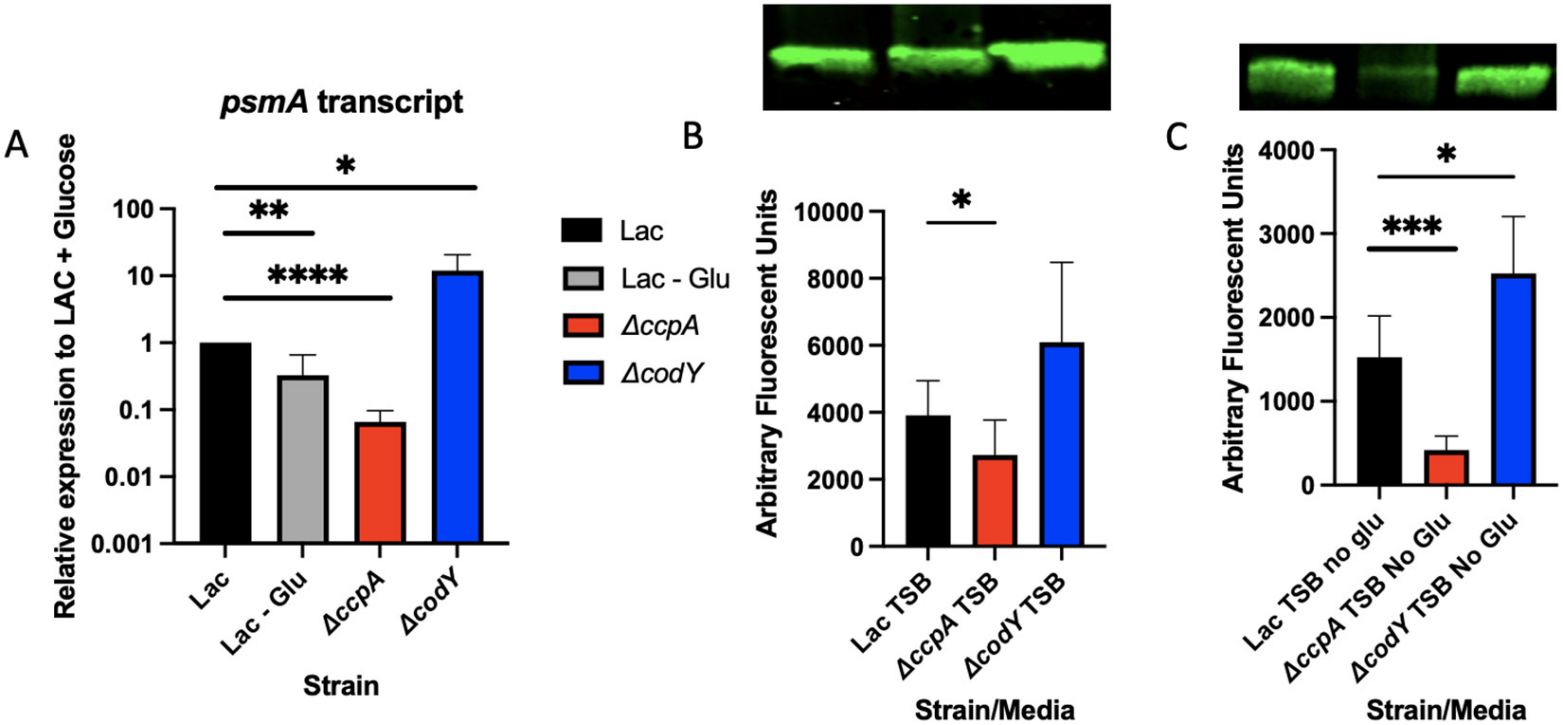
Δ*ccpA* and a Δ*codY* mutants have opposing effects on virulence factor expression. Cultures of WT LAC, *ΔccpA*, and *ΔcodY* were grown to midexponential phase (OD_660_ = ~3–4), and samples were taken for RNA extraction. QRT-PCR was performed on these for expression of *psmA* normalized to *rpoD* (A). The same cultures were also grown overnight in TSB with (B) or without (C) glucose, and their supernatants were run on an SDS-PAGE gel for Western blot stained for α-hemolysin (B, C). Statistics: (A) unpaired *t* test. *, *P* < 0.05; **, *P* < .01; ****, *P* < .0001; (B) paired *t* test. *, *P* < .05; (C) unpaired *t* test. *, *P* < .05; ***, *P* < .001.

The Agr regulon is known to be important for virulence in animals, as it controls the expression of most toxins and proteases in S. aureus, either directly or indirectly (7). We tested the effect of the deletion of *ccpA* on transcription (Fig. 2A) and translation (Fig. 2B and C) of the toxins and proteases affected by Agr. We used RT-PCR to determine the expression level of the phenol-soluble modulin-α (psmα) toxin in WT LAC grown in tryptic soy broth (TSB) with and without glucose, as well as in the *ΔccpA* strain. Expression of psmα is directly controlled by AgrA binding to the psmα promoter, making the expression of these toxins an excellent metric for AgrA activity. We found that the expression of psmα in the *ΔccpA* strain grown in TSB + glucose was reduced to the level of WT LAC grown in TSB − glucose (Fig. 2A). At the protein level, we analyzed the supernatants of overnight culture of WT LAC grown in TSB +/− glucose, as well as *ΔccpA* in TSB +/− glucose, for alpha-toxin levels. Alpha-toxin is one of the most posttranscriptionally regulated proteins by RNAIII, making it a good way to measure the effects of posttranscriptional regulation by the Agr system. We performed a Western blot on the supernatants of WT LAC and *ΔccpA* and found a reduction of alpha-toxin protein in the supernatants of a *ΔccpA* strain in conditions with and without glucose (Fig. 2B and C). These two pieces of data, take together, indicate a reduction of Agr activity in a *ΔccpA* strain.

While the CcpA protein mediates the consumption of glucose, there are other metabolic mediators that can affect the levels of metabolites in bacterial cells. One such regulator is CodY, which represses amino acid synthesis. The CodY regulon would be derepressed in a Δ*codY* strain, possibly resulting in increased production and transport of amino acids. We also investigated the effects of a Δ*codY* mutation on Agr activity, and found, as previously reported, that Agr activity is increased, at both the transcription and translational level (Fig. 2A to C).

### Several amino acids are limited in a *ΔccpA* strain and overabundant in a Δ*codY* strain.

**FIG 3:**
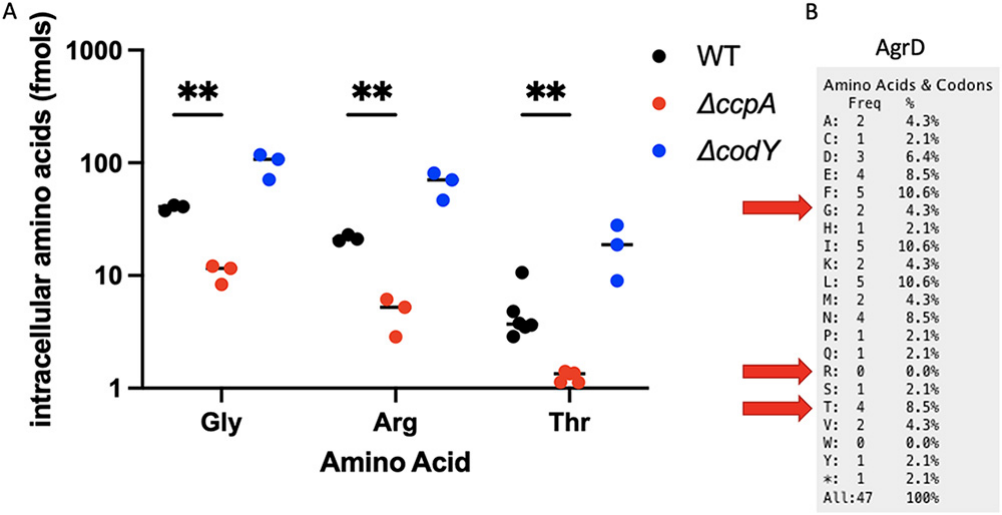
Intracellular levels of key amino acids are altered in a *ΔccpA* and *ΔcodY* mutant. Cell-free extracts were created from cultures of WT LAC, *ΔccpA*, and *ΔcodY* grown to midexponential phase to determine intracellular amino acid levels. Levels of glycine, arginine, and threonine were significantly altered from WT levels in both a *ΔccpA* and a *ΔcodY* mutant (A). Glycine and threonine are both present in the AgrD peptide (B). Statistics: mixed-effects model with Dunnett’s multiple-comparison test. **, *P* < .01.

Knowing that a *ΔccpA* mutant underexpresses and the Δ*codY* mutant overexpresses the Agr regulon, we considered common factors between these two strains. Both mutations are likely to have an impact on amino acid pools, with amino acids being consumed for energy in a *ΔccpA* mutant and excess amino acids being produced/imported by a Δ*codY* mutant. HPLC was used to quantify the amino acid levels present intracellularly in WT LAC and isogenic *ΔccpA* and Δ*codY* mutants. We found elevated levels of all amino acids in a Δ*codY* mutant, compared to WT LAC grown under the same conditions (Fig. S1), but only a few amino acids were significantly reduced in a *ΔccpA* mutant (Fig. 3, S1). These included arginine, threonine, and glycine, all of which are known to be catabolized by S. aureus for energy, especially in a *ΔccpA* mutant (31).

### Amino acid pools and AgrD translation are not responsible for the reduced Agr activity in a Δ*ccpA* strain.

**FIG 4:**
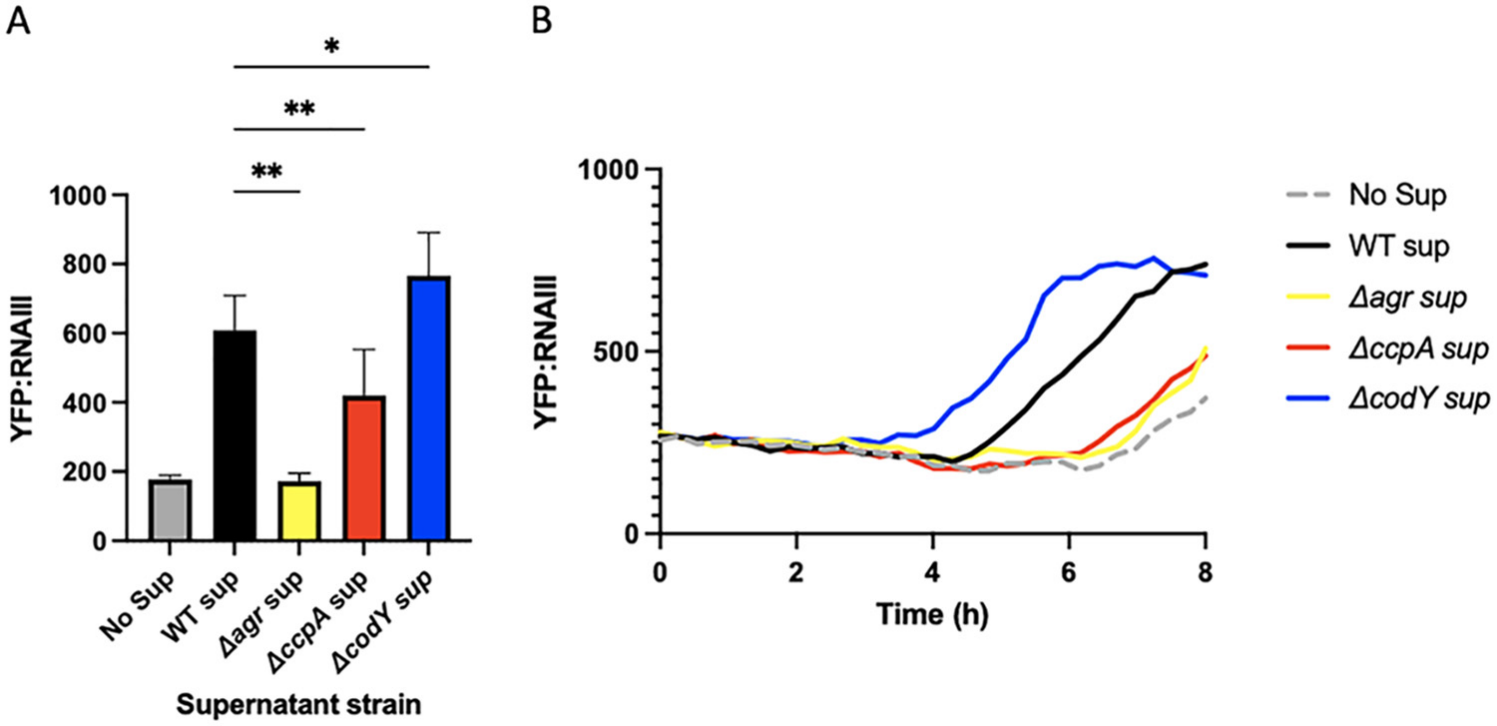
Supernatants from *ΔccpA* and Δ*codY* mutants have opposing effects on the stimulation of an RNAIII::YFP reporter strain. Supernatants were removed from overnight cultures of WT LAC, *ΔccpA*, *ΔcodY*, and *ΔagrA* strains. These were added to fresh cultures of WT LAC harboring a pYFP::RNAIII reporter plasmid. Averages of peak expression levels at hour 6 are shown in A, and a representative curve is shown in B. Statistics: mixed-effect analysis with Tukey’s multiple comparisons. *, *P* < .05; **, *P* < .01.

**FIG 5:**
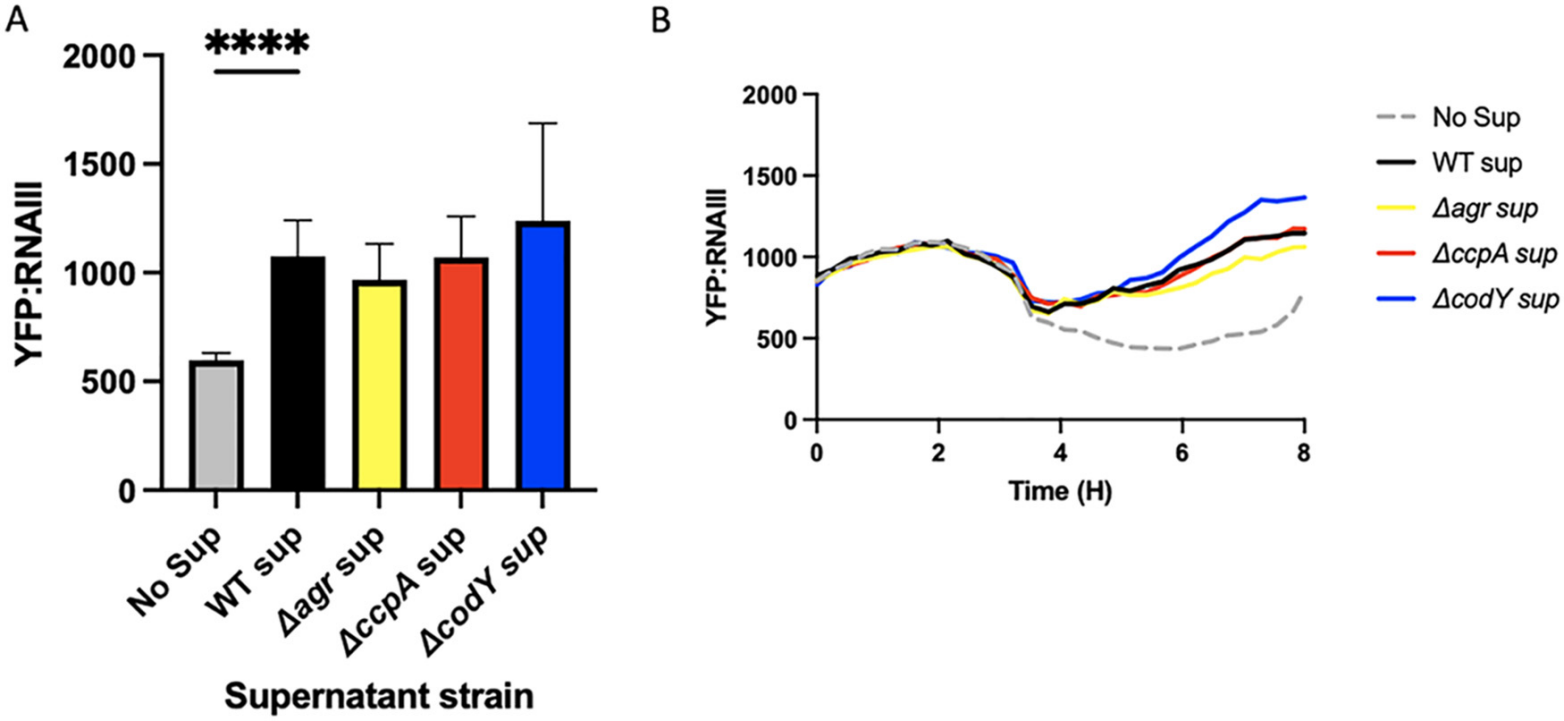
Constitutive expression of AgrBD equalizes RNAIII::YFP stimulation across all mutants. Supernatants were removed from overnight cultures of WT LAC, *ΔccpA*, *ΔcodY*, and *ΔagrA* strains harboring p*lgt::agrBD.* These were added to fresh cultures of WT LAC harboring a pYFP::RNAIII reporter plasmid. Averages of peak expression levels at hour 6 are shown in A, and a representative curve is shown in B. Statistics: mixed-effects model with Dunnett’s multiple-comparison test. ****, *P* < .0001.

Threonine and glycine are both present in the AgrD peptide (Fig. 3). Previous studies have hypothesized that excess amino acid pools in a Δ*codY* mutant could affect the translation of the Agr locus, especially AgrD (18). Increasing the availability of amino acids could allow for the AgrD peptide to be translated more efficiently from its abundant transcript in a quorum-active cell. To test this hypothesis, we used a reporter strain of LAC expressing YFP under the RNAIII promoter (LAC YFP::RNAIII), a promoter directly activated by AgrA, to determine Agr activity. We grew this strain in minimal defined media containing glucose (PNG) and added supernatants from LAC, *ΔccpA*, and Δ*codY* strains grown overnight. If Δ*codY* produced more AgrD peptide than LAC, more AIP would be present in the supernatants of Δ*codY* cultures. Additionally, if the lowered pools of threonine and glycine present intracellularly in a *ΔccpA* strain could impact AgrD translation, lower levels of AIP would be present in these supernatants. Adding these exogenous supernatants prior to quorum activation in the LAC YFP::RNAIII strain will allow the AIP present in these supernatants to induce Agr activity, which will be reflected in YFP levels. YFP levels can be compared to those of a no supernatant control, which will only exhibit YFP induction by its natural Agr system. Differences in YFP production at an early enough time point will indicate differences in exogenously added AIP. As a negative control, we included overnight supernatants from a strain that lacks AgrA, which cannot properly synthesize AIP and exhibits YFP production at the same level as the no supernatant control. Indeed, differences in Agr stimulating capacity of supernatants from mutants were consistent with predicted AIP production in that activity was reduced in Δ*ccpA* and enhanced in Δ*codY* (Fig. 4). However, as the Agr system is positively autoregulated, any impairment in transcription will be observed at the translational level. That is, if the impact of either of these regulators is at the transcriptional level, we would still see a difference in AIP production downstream. Considering this, we cannot be sure that there is a defect in translation of AgrD in a Δ*ccpA* mutant. Attempts to quantify the levels of AgrD transcript resulted in C_t_ values too low to truly determine differences in AgrD transcription. To rectify this issue, we created a plasmid in which the *agrBD* gene is under a constitutive promoter, referred to as p*lgt-agrBD.* By transforming this plasmid into a WT LAC, *ΔccpA*, Δ*codY*, and Δ*agrA* background, we can equilibrate the transcript levels of AgrBD in each of these strains. This allows us to observe the effects of translation specifically on each strain’s supernatant AIP levels when added to the LAC YFP::RNAIII strain in an experiment identical to the above. Upon the introduction of p*lgt-agrBD* into our strains of interest, we observed a similar level of YFP induction in WT, Δ*agrA*, and *ΔccpA* supernatants (Fig. 5). Expressing p*lgt-agrBD* in a Δ*agrA* background is expected to yield similar AIP levels to WT—indeed, there should be no differences in the transcript level or translation of AgrBD in either strain, as AIP production is no longer under Agr control. Surprisingly, the *ΔccpA* strain supernatant also appeared to induce YFP to both WT and Δ*agrA* levels, indicating that there is no translational defect in AgrD production and that the defect in Agr activation seen in a *ΔccpA* mutant is likely at the transcriptional level. The induction of YFP by Δ*codY*, however, trends toward significance (Fig. 5), indicating that there may be increased translation of AgrD transcript by a Δ*codY* mutant, possible due to the excess intracellular amino acids observed in Fig. 3.

### A *ΔccpA* strain has decreased ATP pools in the presence of glucose compared to WT.

**FIG 6:**
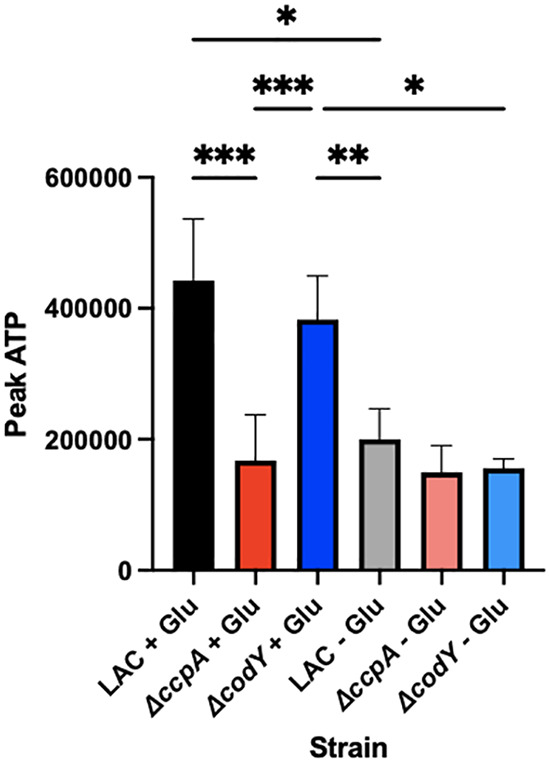
ATP levels in a *ΔccpA* mutant reflect those of WT LAC grown in the absence of glucose. ATP levels were determined in cultures of WT LAC, *ΔccpA*, and *ΔcodY* strains grown in TSB with and without glucose. Statistics: mixed effects model with Tukey’s multiple comparison test. *, *P* < .05; **, *P* < .01; ***, *P* < .001.

To determine what could be having an impact on Agr transcription in a *ΔccpA* mutant, we turned to a hypothesis previously investigated by our lab (30). We have shown that WT S. aureus consuming different carbon sources in minimal defined media has differing levels of ATP and alpha-toxin production (WT LAC grown in chemically defined medium [CDM] with glucose versus casamino acids). As stated previously, because a *ΔccpA* mutant is unable to efficiently consume glucose, it preferentially consumes amino acids. Therefore, we hypothesized that the intracellular ATP levels in a *ΔccpA* mutant would be similar to those in WT LAC grown in the absence of glucose. Quantification of ATP in WT LAC grown in TSB +/− glucose compared to a *ΔccpA* mutant grown in TSB + glucose shows that *ΔccpA* ATP levels are similar to that of WT grown without glucose, despite glucose being present in the media (Fig. 6). Indeed, there appear to be strong similarities between the levels of virulence factor expression as shown in Fig. 2 and the ATP levels demonstrated in Fig. 6. Finally, we see similar ATP levels between WT and Δ*codY* strains, showing that no excess ATP is produced by a Δ*codY* mutant and that ATP levels cannot explain the elevated Agr activity observed by the Δ*codY* mutant.

## DISCUSSION

During infection of a host, the S. aureus bacterium must fight against the host for limited resources to replicate and cause an active infection. While canonically considered nutrients are metals (like iron, zinc, manganese, etc.), this idea of “nutritional immunity” can be expanded to almost any metabolite that a bacterium needs to live—including carbon sources like glucose, amino acids, inorganic phosphate, and so on. A major role of the toxins and proteases so heavily featured when discussing S. aureus virulence factors is to release these important molecules, including amino acids from extracellular proteins broken down by proteases, and nutrients released from host cells lysed by toxins. Considering this role of these virulence factors, logic implies that there should be an impact on their production by metabolic regulators. Indeed, for the past few decades, it has been understood that metabolic transcriptional regulators, such as CcpA and CodY, impact virulence factor production. However, little mechanism for how these regulatory systems interact has been provided.

CcpA plays a key role in carbon catabolite repression, the method that bacteria use to determine if their optimal carbon source is present to consume. For S. aureus, that carbon source is glucose, and the consumption process is glycolysis. CcpA directly activates genes related to glycolysis and directly represses genes related to the consumption of gluconeogenic substrates. Therefore, CcpA activates the optimal carbon catabolism process, which leads to optimized levels of ATP. The ATP from glycolysis itself and additionally from the AckA overflow pathway contribute to increased intracellular ATP concentrations (32). Additionally, as glucose is an important precursor for several macromolecules and essential processes in the cell, in the absence of glucose S. aureus would need to run the process of gluconeogenesis, an energy-expensive process that consumes ATP directly. CcpA also represses this pathway, so in the absence of this important regulator, not only is glycolysis not entirely active, but energy is also being consumed to run gluconeogenesis, even though glucose may be present already in the environment. All this waste of ATP, on top of inefficient ATP generation, leads to the deficit in ATP seen in Fig. 6 between WT LAC growing in the presence and absence of glucose, as well as between the *ΔccpA* mutant and WT. This lowered ATP pool leaves less energy available for other processes, including the synthesis of virulence factors. We propose that the reason these virulence factors are under the control of the Agr system is not simply because of quorum, but because the Agr system is uniquely influenced by the energy state of the cell. The Agr system has a low affinity for ATP (29), and there must be optimal ATP levels in the cell to gain full activation of the Agr system. Energy cannot be wasted on extraneous virulence factors in an energy stressed environment any more than it can be wasted on synthesizing these factors in the absence of quorum sensing. Combining these two influences of the environment into one signaling system that contributes significantly to infection is no doubt evolutionarily important for S. aureus. This lowered affinity for ATP is present in all low GC+ bacteria that possess an Agr-like quorum sensing system, indicating that this specialization is not something that S. aureus has evolved, but rather a larger phenomenon in this family of bacterial species that allows the bacterium to place synthesis of extraneous factors under the control of an energy-optimized system.

CodY is also a transcriptional regulator that reacts to stress from nutrient limitation. Specifically, CodY reacts to lowered levels of BCAAs and GTP. Both of these can become limiting in an infection environment, and the subsequent derepression of the Agr system by CodY could be useful in obtaining the amino acids that act as a stress signal through CodY. However, CodY reacts weakly with the Agr and RNAIII promoter, leading to questions about how CodY affects gene expression. In one of the most thorough studies performed on CodY and Agr to date (18), the authors posit that increased intracellular amino acid pools present via the increased transport and synthesis of amino acids in a Δ*codY* strain could result in the increased translation of the AgrD peptide, which could in turn lead to increased Agr signaling. We showed that the Δ*codY* strain does, in fact, produce more AIP than its WT counterpart. Additionally, while there does seem to be a trend toward increased AIP in the supernatants of a Δ*codY* strain with the autoinduction of the Agr system nullified by constitutive expression of the *agrBD* locus, this trend is not significant. This could be due to the fact that the constitutive expression of *agrBD* under the *lgt* promoter results in enough excess *agrBD* transcript that the differences in amino acids observed in Fig. 3 may not be enough to cause extra translation of these transcripts compared to WT. However, here we can confidently eliminate an effect of Δ*codY* on ATP levels as the means of controlling Agr activity, as a Δ*codY* mutant has increased Agr activity in the presence and absence of glucose despite different intracellular ATP levels (Fig. 6). Further investigation into this hypothesis may be required. We did show, however, that unlike in a *ΔccpA* mutant, deleting Δ*codY* does not affect ATP levels; thus, another explanation for the effect of CodY is needed.

The intersection of metabolism and virulence is a growing area of interest for researchers investigating a number of pathogens, especially pathogens like S. aureus, that are capable of colonizing the host asymptomatically as well as causing devastating disease. The concept of metabolic signals being a trigger for virulence activation has become more popular as researchers realize how important metabolism is to bacterial pathogens. In this study, we are able to shed light on how two different metabolic transcriptional regulators indirectly affect the expression of virulence factors vital for infection. Further study in this field may reveal exactly how important it is for S. aureus to link its survival in the host with its ability to cause infection and metabolize nutrients present underneath the environment it typically colonizes.

## MATERIALS AND METHODS

### Bacterial strains and growth.

The strains used in this study are listed in Table 1. All S. aureus strains are derivatives of USA300 clone LAC. For infection studies, strains were grown in BHI (BD Biosciences). For all other studies, strains were grown in TSB or TSB without glucose (BD Biosciences), or in minimal defined media (PN) with 0.5% glucose (PNG) or 1% amino acids (PNCAA) supplemented as a carbon source (33) at 37°C shaking at 250 RPM with a 10:1 flask:medium ratio to ensure aerobic cultures. The Δ*ccpA* and Δ*codY* mutants were created using homologous recombination and the E. coli harbored PBTK* or PBTT* plasmids, respectively, as previously described (12). Plasmids used in the study were engineered by restriction enzymes in the pOS1 background under the constitutive promoter *lgt.* Antibiotic marked mutations and plasmids were moved into fresh backgrounds using phi-11 phage transduction (34). Antibiotic makers for S. aureus (*E.coli*) are as follows: kanamycin 25 mg/mL, tetracycline 5 mg/mL, chloramphenicol 20 mg/mL, and ampicillin (50 mg/mL).

**TABLE 1:** Strain list

Strain name	Strain description	Source
WT LAC	Methicillin-resistant clinical *S. aureus* isolate	
AR1749	*S. aureus* LAC Δ*ccpA::*Kan	This study
AR1128	*S. aureus* LAC Δ*codY*::Tet	38
AR1198	*S. aureus* LAC Δ*agrA*::Tn	39
AR1038	*S. aureus* LAC pYFP::RNAIII	40
AR1750	*S. aureus* LAC plgt::*agrBD*	This study
AR1751	*S. aureus* LAC Δ*ccpA::*Kan plgt::*agrBD*	This study
AR1752	*S. aureus* LAC Δ*codY*::Tet plgt::*agrBD*	This study
AR1753	*S. aureus* LAC Δ*agrA*::Tn plgt::*agrBD*	This study

### Mouse infections.

C57BL/6J mice of 8–12 weeks of age were intraperitoneally injected with 225 mg/kg dosages of STZ (Sigma-Aldrich). Blood glucose was assayed by glucometer (ReliOn) 3 days after treatment. Only mice with a blood glucose level >300 mg/dL proceeded in the study. Subcutaneous infections were performed as previously described (35). Briefly, bacteria were grown in BHI, washed three times with PBS, plated for enumeration, and stored at 4°C in PBS overnight. Bacteria were diluted to a concentration of 5 × 10^8^, and 20 μL was injected into mice shaved and anesthetized with 2,2,2-tribromoethanol. Mice were monitored daily for weight loss, and at day 7 mice were euthanized with CO_2_ and abscesses were measured. Tissues were collected and homogenized and plated on BHI plates for enumeration. All animal manipulations and infections were conducted under an institutionally approved and currently active IACUC protocol (# 22030607).

### Western blots.

Bacteria for Western blots were grown overnight in TSB with or without dextrose. The following morning 1 mL of culture was centrifuged at 13,000 × *g* for 1 min, the supernatant was removed, and was heat inactivated at 70°C for 10 min. The supernatants were diluted 1:4 with Laemelli buffer (Bio-Rad) with 2-mercaptoethanol (Fischer Scientific) and boiled at 99 C for 10 min. 40 μL of boiled supernatant was loaded onto a Mini Protean TGX 12% gel (Bio-Rad) and run on a Mini Trans-Blot apparatus (Bio-Rad) for 90 min at 150 V with a Chameleon Duo Pre-Stained Protein ladder (LI-COR). The gel was transferred to a supported nitrocellulose 0.22 μm membrane (Bio-Rad) for 60 min at 100 V. The membranes were blocked for 4 h with Intercept protein-free blocking buffer (LI-COR), and then incubated overnight with the Abcam mouse MAb anti-alphahemolysim antibody (ab190467) at 1 μg/mL. The following day, the membranes were washed for 10 min, then 5 min, then 5 min with PBS 0.05% Tween (Sigma-Aldrich). The membranes were then stained with the secondary antibody IR Dye 800CW Donkey anti-mouse (LI-COR) diluted in donkey serum (Sigma-Aldrich) for 1 h, then washed as above. The membranes were imaged on a LI-COR Odyssey machine with 7 (800) and 2 (700) gains on the fluorescent channels.

### RNA extraction and RT-PCR.

RNA extractions were performed as recently described (36). Briefly, ~30 mL of cells was grown in a 250 mL flask, shaking, at 37°C. At a mid-log OD (~4-3), 25 mL of culture was quenched with 50/50 ethanol/acetone at 0°C. The samples were frozen at −80°C. For extraction, the samples were thawed at 30°C and pelleted at 4,250 rpm for 10 min. The supernatant was discarded, and the pellet was air-dried. Upon drying, the pellet was resuspended in 250 μL of Tris-EDTA buffer. The pellet was frozen in a dry ice-ethanol bath and thawed at 60°C three times, and then bead-beat for 1 min and rested on ice for 5 min. We added 650 μL of lysis buffer with 2-mercaptoethanol to the sample and the bead-beating was repeated. The samples were pelleted and 600 μL of supernatant was combined with 600 μL of 70% ethanol. The samples were then processed according to the Invitrogen Pure-link RNA minikit. The purified samples were treated with NEB DNase-1 for 1 h at 37°C and then repurified with the Pure-link RNA minikit.

The RNA samples were quantified, and 50 ng of RNA was added to a RT-PCR according to manufacturer’s instructions for the Power SYBR Green RNA-to C^t^ 1-Step Kit (Applied Biosystems). The machine used was a Bio-Rad iQ5. The C^t^ was determined using the iQ5 software, and the data were analyzed for ΔΔC^t^ based on the control gene *rpoD.* The primers used are listed in Table 2.

**TABLE 2:** Primers

Primer name	Sequence
psma_fwd_RT	TATCAAAAGCTTAATCGAACAATTC
psma_rev_RT	CCCCTTCAAATAAGATGTTCATATC
rpoD_RT.1A	AACTGAATCCAAGTGATCTTAGTG
rpoD_RT.1B	TCATCACCTTGTTCAATACGTTTG
CcpA_3'.1B	GGGGAATTCGTGCCACAATTGGAGGC
CcpA_3'.1A	GGGGAATTCAGGCATTCATCTAACGACCC
CcpA_5'.1A	GGGGGATCCAGCTGGCCGTACGAAAAAGC
CcpA_5'.1B	GGGGGATCCCGCGCTTCTCTTGCTACATC
AgrBD.1A	GGGCATATGAATTATTTTGATAATAAAATTGACCAGTTTGCC
AgrBD.1B	GGGGGATCCTCCACCTACTATCACACTCTC

### Intracellular amino acid assays.

We generated cell extracts (CFEs) from cultures grown to mid-log OD (~3–4), and 50 mL of culture was pelleted at 4,250 RPM for 10 min. The supernatant was discarded, and the cells were resuspended in 5 mL of PBS and then 1 mL of PBS. The 1 mL of cells in PBS were bead-beat for 1 min, then rested on ice for 5 min, three times. The supernatant of this bead-beating was removed and frozen down for analysis.

Samples were thawed and 250 μL was moved into a fresh tube. To remove protein from the sample, 62.5 μL of trichloroacetic acid (Sigma) was added to the CFEs, and the CFEs were incubated on ice with the trichloroacetic acid (TCA) for 10 min. Then the samples were centrifuged at 13,000 × *g* for 10 min. The supernatant from this sample was removed and pH balanced in a new 1.5 mL tube to a pH of 6.2–8. Twenty μL of this pH balanced CFE was derivatized as according to the Waters AccQ-Tag protocol. In short, 20 μL of sample was added to 60 μL of borate buffer, and then 20 μL of “reagent A” was added to the sample. The samples were incubated at 55°C for 10 min, and then diluted 1:10 to a volume of 1 mL. The samples were then run on a Waters alliance HPLC machine as according to the AccQ-Tag protocol. The resulting curves were integrated using the Waters Empower software, and the area under these curves was graphed. The intracellular concentration was determined for significantly different amino acids using standard curves.

### Supernatant stimulation YFP growth curves.

Cultures of LAC, *ΔccpA,* Δ*codY*, *and* Δ*agrA* or each of those strains containing p*lgt::agrBD* were grown to a mid-log OD (~2) in PNG. One mL of cells was removed and pelleted, and the supernatants were filter sterilized through a 0.2 μm filter (Corning). Additionally, overnight cultures of WT LAC harboring the pYFP::RNAIII plasmid (37) were washed three times with PBS and diluted 1:200 in PNG. Two hundred μL of this culture was added to a 96-well flat-bottom plate (Costar), along with 50 μL of the supernatants prepared earlier. A no supernatant control had an additional 50 μL of PNG added. The plates were grown shaking at 37°C in a Bio-Tek Synergy HTX plate reader overnight with readings being taken every 15 min. Curves were analyzed by highlighting the region of time before the no supernatant control showed YFP expression, and examining activation of the wells with each supernatant added. These experiments were repeated in biological and technical triplicate.

### ATP assays.

Cultures of LAC, *ΔccpA,* and Δ*codY* diluted 1:200 from overnight cultures were grown shaking at 37°C in TSB with and without dextrose. Every hour, 100 μL of culture was removed from the culture and added to a 96-well plate containing 100 μL of BacTiter-Glo reagent (Promega). Plates were briefly shaken and then incubated for 5 min. The luminescent signal of each well was determined using a Bio-Tex Synergy plate reader. These experiments were done in technical duplicate and biological triplicate.
