# Supplementary material for: Mechanisms Behind the Indirect Impact of Metabolic Regulators on Virulence Factor Production in Staphylococcus aureus
Source: Microbiol Spectr. 2022 Jul 5;10(4):e02063-22. doi: 10.1128/spectrum.02063-22 (PMC9430575; doi:10.1128/spectrum.02063-22)
Supplement: Supplemental file 1 — Fig. S1. Download spectrum.02063-22-s0001.pdf, PDF file, 0.1 MB [file spectrum.02063-22-s0001.pdf]

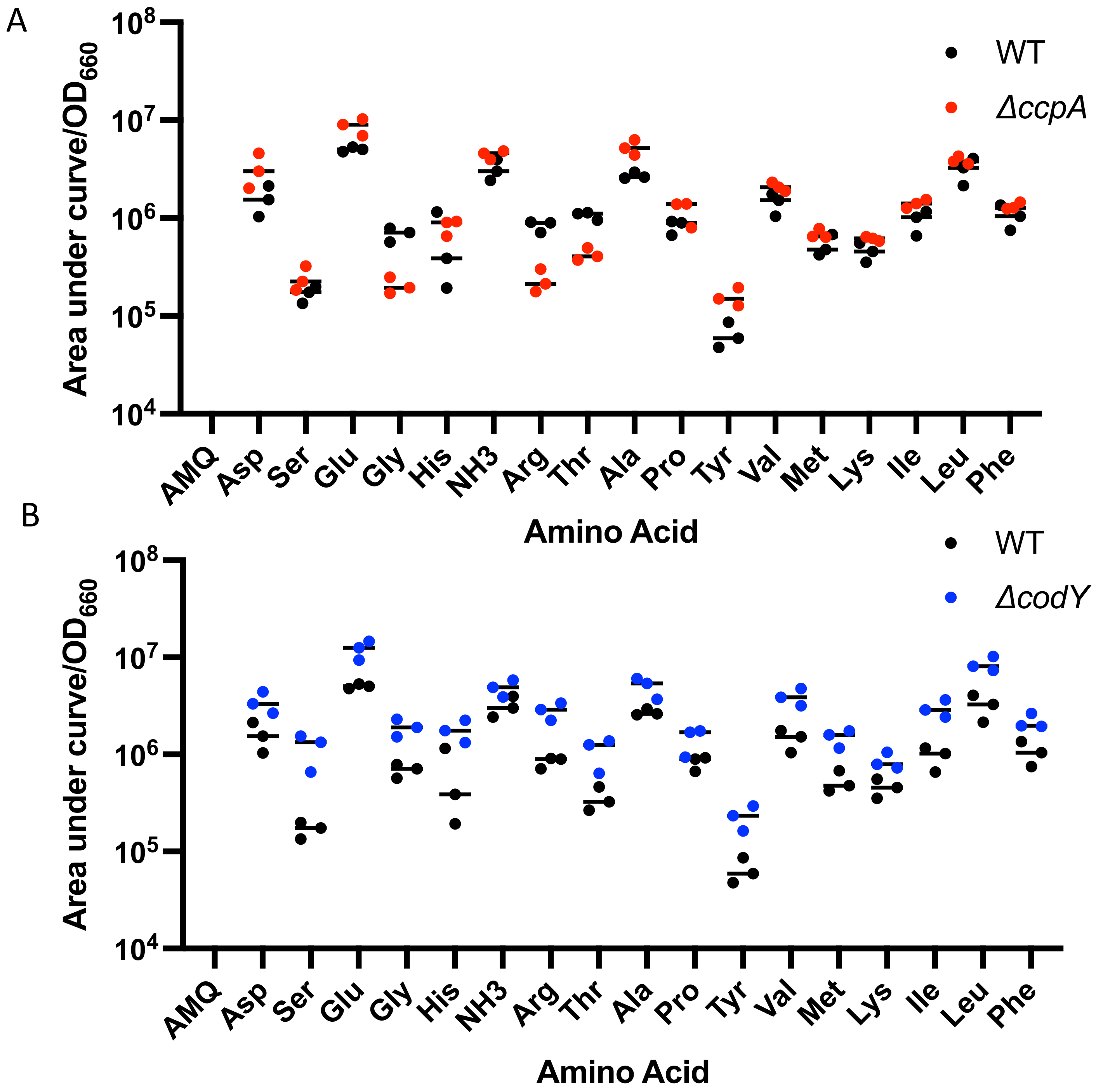

Figure S1: Analysis of intracellular amino acid levels in WT LAC, *ΔccpA* and *ΔcodY* mutants. A few amino acids (R, G and T) are under-represented in a *ΔccpA* strain (A) and all amino acids are significantly over-represented in a *ΔcodY* strain (B). Statistics: 2-way ANOVA with Sidak's multiple comparisons. Significance if $p < .05$
